# Supplementary material for: Rifampicin tolerance and growth fitness among isoniazid-resistant clinical Mycobacterium tuberculosis isolates from a longitudinal study
Source: eLife. 2024 Sep 9;13:RP93243. doi: 10.7554/eLife.93243 (PMC11383526; doi:10.7554/eLife.93243)
Supplement: MDAR checklist [file elife-93243-mdarchecklist1.pdf]

Materials Design Analysis Reporting (MDAR) Checklist for Authors

The MDAR framework establishes a minimum set of requirements in transparent reporting applicable to studies in the life sciences (see Statement of Task: doi:10.31222/osf.oio/9sm4x.). The MDAR checklist is a tool for authors, editors and others seeking to adopt the MDAR framework for transparent reporting in manuscripts and other outputs. Please refer to the MDAR Elaboration Document for additional context for the MDAR framework.

Materials

Antibodies	Yes (indicate where provided: page no/section/legend)	n/a
For commercial reagents, provide supplier name, catalogue number and RRID, if available	No antibodes detected.<br>Please add identifiers for all resources where possible	

Cell Materials	Yes (indicate where provided: page no/section/legend)	n/a
**Cell lines:** Provide species information, strain. Provide accession number in repository OR supplier name, catalog number, clone number, OR RRID	No cell lines detected<br>Please add identifiers for all resources where possible	
**Primary cultures:** Provide species, strain, sex of origin, genetic modification status.	Not currently checked by SciScore	

Experimental Animals	Yes (indicate where provided: page no/section/legend)	n/a
**Laboratory animals:** Provide species, strain, sex, age, genetic modification status. Provide accession number in repository OR supplier name, catalog number, clone number, OR RRID	No organisms detected<br>Please add identifiers for all resources where possible	
**Animal observed in or captured from the field:** Provide species, sex and age where possible	Not currently checked by SciScore	
**Model organisms:** Provide Accession number in repository (where relevant) OR RRID	See laboratory animals section for information.	

Plants and microbes	Yes (indicate where provided: page no/section/legend)	n/a
**Plants:** provide species and strain, unique accession number if available, and source (including location for collected wild specimens)	Not currently checked by SciScore	
**Microbes:** provide species and strain, unique accession number if available, and source	Not currently checked by SciScore	

Human research participants	Yes (indicate where provided: page no/section/legend)	n/a
Identify authority granting ethics approval (IRB or equivalent committee(s), provide reference number for approval.	Methods Ethical approval M. tuberculosis isolates in this study were a part of collection from a previous study25, approved by the Institutional Research Board of Pham Ngoc Thach Hospital as the supervisory institution of the district TB Units (DTUs) in southern Vietnam, Ho Chi Minh City Health Services and the Oxford University Tropical Research Ethics Committee (Oxtrec 030–07).	
Provide statement confirming informed consent obtained from study participants.	Not detected.	
Report on age and sex for all study participants.	**Age:**Briefly, three weeks-old M. tuberculosis colonies from Lowenstein-Jensen medium were used to make cellular suspension in 10 mL saline-Tween80 tube with glass beads (Thermo Fisher, Scientific Inc·, USA) and adjusted to 0.5 McFarland units.<br>**Sex:**not detected.	

Design

Study protocol	Yes (indicate where provided: page no/section/legend)	n/a
For clinical trials, provide the trial registration number OR cite DOI in manuscript.	Not detected.	

Laboratory protocol	Yes (indicate where provided: page no/section/legend)	n/a
Provide DOI or other citation details if detailed step-by-step protocols are available.	Not detected.	

Experimental study design (statistics details)	Yes (indicate where provided: page no/section/legend)	n/a
State whether and how the following have been done, or if they were not carried out		
Sample size determination	not detected.	
Randomization	not detected.	
Blinding	not detected.	
inclusion/exclusion criteria	not detected.	

Sample definition and in-laboratory replication	Yes (indicate where provided: page no/section/legend)	n/a
State number of times the experiment was replicated in laboratory	This washed culture was resuspended in 1mL culture and 100 µL was transferred to 96-well plates as an undiluted culture in duplicate for serial dilution.	
Define whether data describe technical or biological replicates	Not detected.	

Ethics	Yes (indicate where provided: page no/section/legend)	n/a
Studies involving human participants: State details of authority granting ethics approval (IRB or equivalent committee(s), provide reference number for approval.	Methods Ethical approval M. tuberculosis isolates in this study were a part of collection from a previous study25, approved by the Institutional Research Board of Pham Ngoc Thach Hospital as the supervisory institution of the district TB Units (DTUs) in southern Vietnam, Ho Chi Minh City Health Services and the Oxford University Tropical Research Ethics Committee (Oxtrec 030–07).	
Studies involving experimental animals: State details of authority granting ethics approval (IRB or equivalent committee(s), provide reference number for approval.	Not detected.	
Studies involving specimen and field samples: State if relevant permits obtained, provide details of authority approving study; if none were required, explain why.	Not detected.	

Dual Use Research of Concern (DURC)	Yes (indicate where provided: page no/section/legend)	n/a
If study is subject to dual use research of concern, state the authority granting approval and reference number for the regulatory approval	Not currently checked by SciScore	

Analysis

Attrition	Yes (indicate where provided: page no/section/legend)	n/a
State if sample or data point from the analysis is excluded, and whether the criteria for exclusion were determined and specified in advance.	not detected.	

Statistics	Yes (indicate where provided: page no/section/legend)	n/a
Describe statistical tests used and justify choice of tests.	Wilcoxon rank-sum test (stat_compare_means function, ggpubr package) was used to test the null hypothesis that the IS and IR groups have the same continuous distribution, as it is a non-parametric test that does not require a strong assumption about the normality of the distribution of the variable.	

Data availability	Yes (indicate where provided: page no/section/legend)	n/a
State whether newly created datasets are available, including protocols for access or restriction on access.	Not detected.	
If data are publicly available, provide accession number in repository or DOI or URL.	Not detected.	
If publicly available data are reused, provide accession number in repository or DOI or URL, where possible.	Not detected.	

Code availability	Yes (indicate where provided: page no/section/legend)	n/a
For all newly generated code and software essential for replicating the main findings of the study:		
State whether the code or software is available.	Not detected.	
If code is publicly available, provide accession number in repository, or DOI or URL.	Not detected.	

Analysis

Adherence to community standards	Yes (indicate where provided: page no/section/legend)	n/a
MDAR framework recommends adoption of discipline-specific guidelines, established and endorsed through community initiatives. Journals have their own policy about requiring specific guidelines and recommendations to complement MDAR.		
State if relevant guidelines (eg., ICMJE, MIBBI, ARRIVE) have been followed, and whether a checklist (eg., CONSORT, PRISMA, ARRIVE) is provided with the manuscript.	Not currently checked by SciScore	
