# Supplementary material for: Rifampicin tolerance and growth fitness among isoniazid-resistant clinical Mycobacterium tuberculosis isolates from a longitudinal study
Source: eLife. 2024 Sep 9;13:RP93243. doi: 10.7554/eLife.93243 (PMC11383526; doi:10.7554/eLife.93243)
Supplement: Supplementary file 1. [file elife-93243-supp1.docx]

**Supplementary file 1**

**Supplementary file 1a**

| Number of  *M. tuberculosis* isolates | Rifampicin MIC | Rifampicin 2 µg/mL as times of MIC |
| --- | --- | --- |
| 4 | 0.03 | 66.6 |
| 104 | 0.06 | 33.3 |
| 55 | 0.12 | 16.6 |
| 20 | 0.25 | 8.0 |
| 3 | 0.5 | 4.0 |

**Supplementary file 1b**

|  |  |  |  |  |  |  |  |  |  |  |  |  |  |  |
| --- | --- | --- | --- | --- | --- | --- | --- | --- | --- | --- | --- | --- | --- | --- |
| Patient number | trend_group_D5_15 | trend_group_D5_60 | *M. tb* isolate collection  (in months) | Compari-sion between two isolates | SNP differe-nce | isolate 1 | isolate 2 | amino_  acid change | Locus_gene | product | gene | locus_tag | Repetitive-e_region |  |
| 1 | Increase | Increase | 0M, 1M, 8M | 0M, 1M | 0 | NA | ad_ratio:0.9 | E52G | Rv1793 (esxN) | ESAT-6 like protein EsxN | esxN | Rv1793 | FALSE |  |
| 1 | Increase | Increase | 0M, 1M, 8M | 0M, 1M | 0 | NA | ad_ratio:0.9 | N38S | Rv2543 (lppA) | lipoprotein LppA | lppA | Rv2543 | FALSE |  |
| 1 | Increase | Increase | 0M, 1M, 8M | 0M, 1M | 0 | NA | ad_ratio:0.9 | R278H | Rv3138 (pflA) | pyruvate formate lyase activating protein PflA | pflA | Rv3138 | FALSE |  |
| 1 | Increase | Increase | 0M, 1M, 8M | 0M, 1M | 0 | ad_ratio:0.9 | NA | R389W | Rv1319c (NA) | adenylate cyclase | NA | Rv1319c | FALSE |  |
| 2 | Increase | Decrease | 0M, 1M, 12M | 0M, 1M | 0 | NA | ad_ratio:0.9 | H37Q | Rv2543 (lppA) | lipoprotein LppA | lppA | Rv2543 | FALSE |  |
| 2 | Increase | Decrease | 0M, 1M, 12M | 0M, 1M | 0 | NA | ad_ratio:0.9 | N38S | Rv2543 (lppA) | lipoprotein LppA | lppA | Rv2543 | FALSE |  |
| 3 | Increase | Unchange | 0M, 1M | 0M, 1M | 3 | NA | ad_ratio:0.9 | G407V | Rv1266c (pknH) | serine/threonine-protein kinase PknH | pknH | Rv1266c | FALSE |  |
| 3 | Increase | Unchange | 0M, 1M | 0M, 1M | 3 | NA | ad_ratio:0.9 | L404F | Rv1266c (pknH) | serine/threonine-protein kinase PknH | pknH | Rv1266c | FALSE |  |
| 3 | Increase | Unchange | 0M, 1M | 0M, 1M | 3 | NA | ad_ratio:0.9 | G174R | Rv2351c (plcA) | membrane-associated phospholipase A | plcA | Rv2351c | FALSE |  |
| 3 | Increase | Unchange | 0M, 1M | 0M, 1M | 3 | NA | ad_ratio:0.9 | I171V | Rv2351c (plcA) | membrane-associated phospholipase A | plcA | Rv2351c | FALSE |  |
| 3 | Increase | Unchange | 0M, 1M | 0M, 1M | 3 | NA | ad_ratio:0.9 | T168A | Rv2351c (plcA) | membrane-associated phospholipase A | plcA | Rv2351c | FALSE |  |
| 3 | Increase | Unchange | 0M, 1M | 0M, 1M | 3 | NA | PASS | P5L | Rv2802c (NA) | arginine/hypothetical protein | NA | Rv2802c | FALSE |  |
| 3 | Increase | Unchange | 0M, 1M | 0M, 1M | 3 | NA | ad_ratio:0.9 | A38G | Rv3901c (NA) | membrane protein | NA | Rv3901c | FALSE |  |
| 3 | Increase | Unchange | 0M, 1M | 0M, 1M | 3 | NA | ad_ratio:0.9 | T16I | Rv3901c (NA) | membrane protein | NA | Rv3901c | FALSE |  |
| 3 | Increase | Unchange | 0M, 1M | 0M, 1M | 3 | ad_ratio:0.9 | NA | R6G | Rv1758 (cut1) | cutinase | cut1 | Rv1758 | FALSE |  |
| 3 | Increase | Unchange | 0M, 1M | 0M, 1M | 3 | PASS | NA | I463S | Rv2488c (NA) | LuxR family transcriptional regulator | NA | Rv2488c | FALSE |  |
| 3 | Increase | Unchange | 0M, 1M | 0M, 1M | 3 | PASS | NA | S128W | Rv2828c (NA) | hypothetical protein | NA | Rv2828c | FALSE |  |
| 4 | Increase | Increase | 0M, 1M, 2M, 12M, | 0M, 12M | 0 | NA | ad_ratio:0.9 | T178A | Rv0792c (NA) | transcriptional regulator | NA | Rv0792c | FALSE |  |
| 4 | Increase | Increase | 0M, 1M, 2M, 12M, | 0M, 12M | 0 | NA | ad_ratio:0.9 | D48E | Rv1907c (NA) | hypothetical protein | NA | Rv1907c | FALSE |  |
| 4 | Increase | Increase | 0M, 1M, 2M, 12M, | 0M, 12M | 0 | NA | ad_ratio:0.9 | V96A | Rv3424c (NA) | hypothetical protein | NA | Rv3424c | FALSE |  |
| 4 | Increase | Increase | 0M, 1M, 2M, 12M, | 0M, 12M | 0 | ad_ratio:0.9 | NA | R389W | Rv1319c (NA) | adenylate cyclase | NA | Rv1319c | FALSE |  |
| 4 | Increase | Increase | 0M, 1M, 2M, 12M, | 0M, 12M | 0 | ad_ratio:0.9 | NA | N378D | Rv3680 (NA) | anion transporter ATPase | NA | Rv3680 | FALSE |  |
| 5 | Increase | Decrease | 0M, 24M | 0M, 24M | 3 | NA | PASS | L69F | Rv2329c (narK1) | nitrate/nitrite transporter | narK1 | Rv2329c | FALSE |  |
| 5 | Increase | Decrease | 0M, 24M | 0M, 24M | 3 | ad_ratio:0.9 | NA | R389W | Rv1319c (NA) | adenylate cyclase | NA | Rv1319c | FALSE |  |
| 7 | Increase | Decrease | 0M, 1M, 18M | 0M, 1M | 0 | NA | ad_ratio:0.9 | V96A | Rv3424c (NA) | hypothetical protein | NA | Rv3424c | FALSE |  |
| 8 | Increase | Increase | 0M, 2M | 0M, 2M | 0 | NA | ad_ratio:0.9 | D48E | Rv1907c (NA) | hypothetical protein | NA | Rv1907c | FALSE |  |
| 9 | Increase | Increase | 0M, 1M, 12M | 0M, 12M | 3 | NA | PASS | R566H | Rv0973c (accA2) | acetyl/propionyl-CoA carboxylase subuit alpha | accA2 | Rv0973c | FALSE |  |
| 9 | Increase | Increase | 0M, 1M, 12M | 0M, 12M | 3 | NA | PASS | W205* | Rv1270c (lprA) | lipoprotein LprA | lprA | Rv1270c | FALSE |  |
| 9 | Increase | Increase | 0M, 1M, 12M | 0M, 12M | 3 | NA | ad_ratio:0.9 | D439E | Rv1319c (NA) | adenylate cyclase | NA | Rv1319c | FALSE |  |
| 9 | Increase | Increase | 0M, 1M, 12M | 0M, 12M | 3 | NA | PASS | D191G | Rv3483c (NA) | hypothetical protein | NA | Rv3483c | FALSE |  |
| 12 | Unchange | Increase | 0M, 8M | 0M, 8M | 1 | ad_ratio:0.9 | NA | R389W | Rv1319c (NA) | adenylate cyclase | NA | Rv1319c | FALSE |  |
| 12 | Unchange | Increase | 0M, 8M | 0M, 8M | 1 | ad_ratio:0.9 | NA | N378D | Rv3680 (NA) | anion transporter ATPase | NA | Rv3680 | FALSE |  |
| 13 | Unchange | Unchange | 0M, 12M | 0M, 12M | 3 | NA | PASS | Q126K | Rv2083 (NA) | hypothetical protein | NA | Rv2083 | FALSE |  |
| 13 | Unchange | Unchange | 0M, 12M | 0M, 12M | 3 | ad_ratio:0.9 | NA | R389W | Rv1319c (NA) | adenylate cyclase | NA | Rv1319c | FALSE |  |
| 13 | Unchange | Unchange | 0M, 12M | 0M, 12M | 3 | ad_ratio:0.9 | NA | G323S | Rv2318 (uspC) | sugar ABC transporter substrate-binding lipoprotein UspC | uspC | Rv2318 | FALSE |  |
| 13 | Unchange | Unchange | 0M, 12M | 0M, 12M | 3 | PASS | NA | G191R | Rv2836c (dinF) | DNA-damage-inducible protein DinF | dinF | Rv2836c | FALSE |  |
| 13 | Unchange | Unchange | 0M, 12M | 0M, 12M | 3 | PASS | NA | V164M | Rv2893 (NA) | oxidoreductase | NA | Rv2893 | FALSE |  |
| 14 | Decrease | Decrease | 0M, 18M | 0M, 18M | 3 | NA | ad_ratio:0.9 | G292S | Rv1997 (ctpF) | cation transporter ATPase F | ctpF | Rv1997 | FALSE |  |
| 14 | Decrease | Decrease | 0M, 18M | 0M, 18M | 3 | NA | PASS | H347Y | Rv2394 (ggtB) | gamma-glutamyltranspeptidase precursor GgtB | ggtB | Rv2394 | FALSE |  |
| 14 | Decrease | Decrease | 0M, 18M | 0M, 18M | 3 | NA | PASS | P188S | Rv2728c (NA) | hypothetical protein | NA | Rv2728c | FALSE |  |
| 14 | Decrease | Decrease | 0M, 18M | 0M, 18M | 3 | PASS | NA | W12R | Rv1899c (lppD) | lipoprotein LppD | lppD | Rv1899c | FALSE |  |
| 16 | Decrease | Increase | 0M, 5M | 0M, 5M | 2 | NA | PASS | M382I | Rv1704c (cycA) | D-serine/alanine/glycine transporter protein CycA | cycA | Rv1704c | FALSE |  |
| 16 | Decrease | Increase | 0M, 5M | 0M, 5M | 2 | NA | ad_ratio:0.9 | V73A | Rv1883c (NA) | hypothetical protein | NA | Rv1883c | FALSE |  |
| 16 | Decrease | Increase | 0M, 5M | 0M, 5M | 2 | ad_ratio:0.9 | NA | I171V | Rv2351c (plcA) | membrane-associated phospholipase A | plcA | Rv2351c | FALSE |  |
| 16 | Decrease | Increase | 0M, 5M | 0M, 5M | 2 | ad_ratio:0.9 | NA | T168A | Rv2351c (plcA) | membrane-associated phospholipase A | plcA | Rv2351c | FALSE |  |
| 16 | Decrease | Increase | 0M, 5M | 0M, 5M | 2 | ad_ratio:0.9 | NA | A139T | Rv2543 (lppA) | lipoprotein LppA | lppA | Rv2543 | FALSE |  |
| 17 | Decrease | Increase | 1M, 8M | 1M, 8M | 11 | NA | PASS | Y1638H | Rv0101 (nrp) | peptide synthetase Nrp | nrp | Rv0101 | FALSE |  |
| 17 | Decrease | Increase | 1M, 8M | 1M, 8M | 11 | NA | PASS | R384W | Rv1696 (recN) | DNA repair protein RecN | recN | Rv1696 | FALSE |  |
| 17 | Decrease | Increase | 1M, 8M | 1M, 8M | 11 | NA | PASS | Q10* | Rv2043c (pncA) | pyrazinamidase/nicotinamidase PncA | pncA | Rv2043c | FALSE |  |
| 17 | Decrease | Increase | 1M, 8M | 1M, 8M | 11 | NA | PASS | K342E | Rv2400c (subI) | sulfate ABC transporter substrate-binding lipoprotein SubI | subI | Rv2400c | FALSE |  |
| 17 | Decrease | Increase | 1M, 8M | 1M, 8M | 11 | NA | PASS | P43R | Rv2544 (lppB) | lipoprotein LppB | lppB | Rv2544 | FALSE |  |
| 17 | Decrease | Increase | 1M, 8M | 1M, 8M | 11 | NA | PASS | H44R | Rv2544 (lppB) | lipoprotein LppB | lppB | Rv2544 | FALSE |  |
| 17 | Decrease | Increase | 1M, 8M | 1M, 8M | 11 | NA | ad_ratio:0.9 | L33F | Rv2545 (vapB18) | antitoxin VapB18 | vapB18 | Rv2545 | FALSE |  |
| 17 | Decrease | Increase | 1M, 8M | 1M, 8M | 11 | NA | PASS | P335L | Rv2689c (NA) | hypothetical protein | NA | Rv2689c | FALSE |  |
| 17 | Decrease | Increase | 1M, 8M | 1M, 8M | 11 | ad_ratio:0.9 | NA | V73A | Rv1883c (NA) | hypothetical protein | NA | Rv1883c | FALSE |  |
| 17 | Decrease | Increase | 1M, 8M | 1M, 8M | 11 | PASS | NA | L80P | Rv2398c (cysW) | sulfate ABC transporter permease CysW | cysW | Rv2398c | FALSE |  |
| 18 | Decrease | Increase | 0M, 12M, 18M | 0M, 18M | 3 | NA | PASS | F209L | Rv3758c (proV) | glycine betaine/carnitine/choline/L-proline ABC transporter ATP-binding protein ProV | proV | Rv3758c | FALSE |  |

NA - Wild-type

ad_ratio:0.9 - Emerging Non-synonymous SNP in reads below 90% threshold.

Pass - Emerging Non-synonymous SNP in reads above 90% threshold.

Note: SNPs difference include both synonymous and nonsynonymous variants (only Pass).

**Supplementary file 1c**

| Gene name | Mutation | Gene function | Associated change rifampicin tolerance – D5-15 | Associated change rifampicin tolerance – D5-60 | Reported function related to survival or antibiotic response | Reference |
| --- | --- | --- | --- | --- | --- | --- |
| Rv0101 | Y1638H | Probable peptide synthetase nrp | Decrease | Increase | - Down regulated upon rifampicin exposure of MDR-H37Rv - Prevented INH-mediated killing *M. tuberculosis* in Mice | Knegt et. al., 2013  Dhar and McKinney 2010 |
| Rv0792c | T178A | Probable transcription regulatory protein (gntr family) | Increase | Increase | Gene upregulated in *M. tuberculosis* persisters. | Keren et. al., 2011 |
| Rv0973c | R566H | Lipid metabolism | Increase | Increase | Gene downregulated upon HigB toxin expression in *M. tuberculosis* | Schuessler et. al., 2013 |
| Rv1266c | G407V | *pknH*, Serine/threonine protein kinase | Increase | No change | Interaction partner of FtsB in *M. tuberculosis,* regulation of cell division during persistence. | Wang et. al., 2019 |
|  | L404F |  | Increase | No change |  |  |
| Rv1270c | W205* | *lprA*, Cell wall and cell processes | Increase | Increase | Gene upregulated upon *sigF* induction | Williams et. al., 2007 |
| Rv1319c | D439E | Putative adenylate cyclase, regulation of cellular metabolism | Increase | Increase | Gene displaying high within-host genetic diversity. | Nimmo et. al., 2020 |
|  | R389W |  | Increase  (n = 3),  No change (n = 2), Decrease  (n = 1). | Increase  (n = 3),  no change (n = 1), decrease  (n = 1). |  |  |
| Rv1696 | R384W | *RccN*, works in DNA repair | Decrease | Increase | DNA repair mechanism in *M. tuberculosis* | Mittal et. al., 2020 |
| Rv1704c | M382I | Alanine transporter CycA | Decrease | Increase | Gene involved in D-cycloserine resistance | Vilcheze 2020 |
| Rv1758 | R6G |  | Increase | Decrease | Gene frameshift deletion associated with hypervirulence and enhanced growth in macrophage. | Lam et. al., 2011 |
| Rv1883c | V73A |  | Decrease  (n = 2) | Increase  (n = 2) | Known mutation associated with IR | Lagutkin et. al., 2022 |
| Rv1899c | W12R |  | Decrease | Decrease | No report |  |
| Rv1907c | D48E |  | Increase  (n = 2) | Increase  (n = 2) | Gene associated with IR | Lagutkin et. al., 2022 |
| Rv1997 | G292S | *ctpF M. tuberculosis* cation transporter | Decrease | Decrease | Gene regulated by DosR, involved in hypoxia, dormancy regulon and ion transport | Pulido et. al., 2014 |
| Rv2043c | Q10* | *pncA,* pyrazinamidase enzyme | Decrease | Increase | Gene involved in Pyrazinamide resistance | Baddam et. al., 2018 |
| Rv2083 | Q126K |  | No change | No change |  |  |
| Rv2318 | G323S |  | No change | No change |  |  |
| Rv2329c | L69F | *narK1* | Increase | Decrease | Gene overexpressed under nitrogen limitation in *M. tuberculosis* | Williams et. al., 2015 |
| Rv2351c | G174R | *plcA* Membrane-associated phospholipase C | Increase | Decrease | Gene displaying high within-host genetic diversity. | Shockey et. al., 2019 |
|  | I171V |  | Increase  (n = 1)  Decrease  (n = 1) | Increase  (n = 1)  No change  (n = 1) |  |  |
|  | T168A |  | Increase  (n = 1)  Decrease  (n = 1) | Increase  (n = 1)  No change  (n = 1) |  |  |
| Rv2394 | H347Y | *ggtB*, Oxidative stress proteins | Decrease | Decrease | Gene upregulated in *M. tuberculosis* under oxidative stress induced by sulfamethoxazole | Sarkar et. al., 2018 |
| Rv2398c | L80P | *cysW*, Sulphate transport system permease protein, resuscitation-promoting factor. | Decrease | Increase | Gene upregulated under starvation model of *M. tuberculosis* persistence | Betts et. al., 2002  Gorla et. al., 2018 |
| Rv2400c | K342E | *subI* Sulphate binding precursor | Decrease | Increase | - Gene upregulated under starvation model of *M. tuberculosis* persistence - Possible role in antibiotic tolerance. | Betts et. al., 2002  Xu et. al., 2017 |
| Rv2488c | I463S | LuxR family regulator | Increase | Decrease | Gene family involved in *M. tuberculosis* dormancy and virulence | Fang et. al., 2013 |
| Rv2543 | A139T | *IppA* | Decrease | Increase | Regulated by stationary phase sigma factor *sigD* | Calamita et. al., 2005 |
|  | H37Q |  | Increase | Decrease |  |  |
|  | N38S |  | Increase  (n = 2) | Increase  (n = 1)  Decrease  (n = 1) |  |  |
| Rv2544 | H44R | Membrane lipoprotein | Decrease | Decrease |  | Fleischmann et. al., 2002 |
|  | P43R |  | Increase | Increase |  |  |
| Rv2545 | L33F | *vapBC* Toxin-antitoxin modules | Decrease | Increase | Regulate bacterial growth arrest. | Ahidjo et. al., 2011 |
| Rv2689c | P335L |  | Decrease | Increase | Gene with mutation during *M. tuberculosis* latent infection | Colangeli et. al., 2014 |
| Rv2728c | P188S | Conserved alanine rich protein | Decrease | Decrease |  | Li et. al., 2015 |
| Rv2836c | G191R | *dinF* | No change | No change | Gene associated with DNA repair and upregulated in pulmonary TB | Rachman et. al., 2006 |
| Rv2893 | V164M | Similar to alkanal monooxygenase alpha chain | No change | No change | Gene repressed in acid stress | Fisher et. al., 2002 |
| Rv3138 | R278H | Pyruvate–formate–lyase activating protein | Increase | Increase | Gene upregulated in *M. tuberculosis* stationary phase | Hampshire et. al., 2004 |
| Rv3424c | V96A |  | Increase  (n = 2) | Increase  (n = 1)  Decrease  (n = 1) | Gene displaying high within-host genetic diversity. | Nimmo et. al., 2020 |
| Rv3483c | D191G |  | Increase | Increase | MmpL3 (mycolic acid transporter) interacting protein | Belardinelli et. al., 2019 |
| Rv3680 | N378D |  | Increase  (n = 1)  No change  (n = 1) | Increase  (n = 2) | Gene involved in protecting *M. tuberculosis* from Glycerol and Nitric Oxide toxicity | Whitaker et. al., 2020 |
| Rv3758c | F209L | *proV* | Decrease | Increase | Role in the maintenance of osmoregulation within the phagosome | Gautam et. al., 2019 |
| Rv3901c | A38G |  | Increase | No change | Role in virulence of *M. marinum* | Ruley et. al., 2004 |
|  | T16I |  | Increase | No change |  |  |

References for the supplementary table 3.

1. de Knegt GJ, Bruning O, ten Kate MT, et al. Rifampicin-induced transcriptome response in rifampicin-resistant Mycobacterium tuberculosis. Tuberculosis (Edinb) 2013; 93(1): 96-101.

2. Dhar N, McKinney JD. Mycobacterium tuberculosis persistence mutants identified by screening in isoniazid-treated mice. Proc Natl Acad Sci U S A 2010; 107(27): 12275-80.

3. Keren I, Minami S, Rubin E, Lewis K. Characterization and transcriptome analysis of Mycobacterium tuberculosis persisters. mBio 2011; 2(3): e00100-11.

4. Schuessler DL, Cortes T, Fivian-Hughes AS, et al. Induced ectopic expression of HigB toxin in Mycobacterium tuberculosis results in growth inhibition, reduced abundance of a subset of mRNAs and cleavage of tmRNA. Mol Microbiol 2013; 90(1): 195-207.

5. Wang R, Kreutzfeldt K, Botella H, Vaubourgeix J, Schnappinger D, Ehrt S. Persistent Mycobacterium tuberculosis infection in mice requires PerM for successful cell division. Elife 2019; 8.

6. Williams EP, Lee JH, Bishai WR, Colantuoni C, Karakousis PC. Mycobacterium tuberculosis SigF regulates genes encoding cell wall-associated proteins and directly regulates the transcriptional regulatory gene phoY1. J Bacteriol 2007; 189(11): 4234-42.

7. Nimmo C, Brien K, Millard J, et al. Dynamics of within-host Mycobacterium tuberculosis diversity and heteroresistance during treatment. EBioMedicine 2020; 55: 102747.

8. Mittal P, Sinha R, Kumar A, et al. Focusing on DNA Repair and Damage Tolerance Mechanisms in Mycobacterium tuberculosis: An Emerging Therapeutic Theme. Curr Top Med Chem 2020; 20(5): 390-408.

9. Lam JT, Yuen KY, Ho PL, et al. Truncated Rv2820c enhances mycobacterial virulence ex vivo and in vivo. Microb Pathog 2011; 50(6): 331-5.

10. Lagutkin D, Panova A, Vinokurov A, Gracheva A, Samoilova A, Vasilyeva I. Genome-Wide Study of Drug Resistant Mycobacterium tuberculosis and Its Intra-Host Evolution during Treatment. Microorganisms 2022; 10(7).

11. Pulido PA, Novoa-Aponte L, Villamil N, Soto CY. The DosR dormancy regulator of Mycobacterium tuberculosis stimulates the Na(+)/K (+) and Ca (2+) ATPase activities in plasma membrane vesicles of mycobacteria. Curr Microbiol 2014; 69(5): 604-10.

12. Baddam R, Kumar N, Wieler LH, et al. Analysis of mutations in pncA reveals non-overlapping patterns among various lineages of Mycobacterium tuberculosis. Sci Rep 2018; 8(1): 4628.

13. Williams KJ, Jenkins VA, Barton GR, Bryant WA, Krishnan N, Robertson BD. Deciphering the metabolic response of Mycobacterium tuberculosis to nitrogen stress. Mol Microbiol 2015; 97(6): 1142-57.

14. Shockey AC, Dabney J, Pepperell CS. Effects of Host, Sample, and in vitro Culture on Genomic Diversity of Pathogenic Mycobacteria. Front Genet 2019; 10: 477.

15. Sarkar R, Mdladla C, Macingwana L, et al. Proteomic analysis reveals that sulfamethoxazole induces oxidative stress in M. tuberculosis. Tuberculosis (Edinb) 2018; 111: 78-85.

16. Betts JC, Lukey PT, Robb LC, McAdam RA, Duncan K. Evaluation of a nutrient starvation model of Mycobacterium tuberculosis persistence by gene and protein expression profiling. Mol Microbiol 2002; 43(3): 717-31.

17. Xu W, DeJesus MA, Rucker N, et al. Chemical Genetic Interaction Profiling Reveals Determinants of Intrinsic Antibiotic Resistance in Mycobacterium tuberculosis. Antimicrob Agents Chemother 2017; 61(12).

18. Fang H, Yu D, Hong Y, Zhou X, Li C, Sun B. The LuxR family regulator Rv0195 modulates Mycobacterium tuberculosis dormancy and virulence. Tuberculosis (Edinb) 2013; 93(4): 425-31.

19. Calamita H, Ko C, Tyagi S, Yoshimatsu T, Morrison NE, Bishai WR. The Mycobacterium tuberculosis SigD sigma factor controls the expression of ribosome-associated gene products in stationary phase and is required for full virulence. Cell Microbiol 2005; 7(2): 233-44.

20. Fleischmann RD, Alland D, Eisen JA, et al. Whole-genome comparison of Mycobacterium tuberculosis clinical and laboratory strains. J Bacteriol 2002; 184(19): 5479-90.

21. Ahidjo BA, Kuhnert D, McKenzie JL, et al. VapC toxins from Mycobacterium tuberculosis are ribonucleases that differentially inhibit growth and are neutralized by cognate VapB antitoxins. PLoS One 2011; 6(6): e21738.

22. Colangeli R, Arcus VL, Cursons RT, et al. Whole genome sequencing of Mycobacterium tuberculosis reveals slow growth and low mutation rates during latent infections in humans. PLoS One 2014; 9(3): e91024.

23. Li W, Fan X, Long Q, Xie L, Xie J. Mycobacterium tuberculosis effectors involved in host-pathogen interaction revealed by a multiple scales integrative pipeline. Infect Genet Evol 2015; 32: 1-11.

24. Rachman H, Strong M, Ulrichs T, et al. Unique transcriptome signature of Mycobacterium tuberculosis in pulmonary tuberculosis. Infect Immun 2006; 74(2): 1233-42.

25. Fisher MA, Plikaytis BB, Shinnick TM. Microarray analysis of the Mycobacterium tuberculosis transcriptional response to the acidic conditions found in phagosomes. J Bacteriol 2002; 184(14): 4025-32.

26. Hampshire T, Soneji S, Bacon J, et al. Stationary phase gene expression of Mycobacterium tuberculosis following a progressive nutrient depletion: a model for persistent organisms? Tuberculosis (Edinb) 2004; 84(3-4): 228-38.

27. Belardinelli JM, Stevens CM, Li W, et al. The MmpL3 interactome reveals a complex crosstalk between cell envelope biosynthesis and cell elongation and division in mycobacteria. Sci Rep 2019; 9(1): 10728.

28. Whitaker M, Ruecker N, Hartman T, et al. Two interacting ATPases protect Mycobacterium tuberculosis from glycerol and nitric oxide toxicity. J Bacteriol 2020; 202(16).

29. Gautam US, Mehra S, Kumari P, et al. Mycobacterium tuberculosis sensor kinase DosS modulates the autophagosome in a DosR-independent manner. Commun Biol 2019; 2: 349.

30. Ruley KM, Ansede JH, Pritchett CL, Talaat AM, Reimschuessel R, Trucksis M. Identification of Mycobacterium marinum virulence genes using signature-tagged mutagenesis and the goldfish model of mycobacterial pathogenesis. FEMS Microbiol Lett 2004; 232(1): 75-81.

31. Catherine Vilchèze Mycobacterial Cell Wall: A Source of Successful Targets for Old and New Drugs http://dx.doi.org/10.3390/app10072278
